# Dysentery

**Published:** 1848-11

**Authors:** 


*Dysentery.*—To THE EDITOR. Sir,—I have recently used the following mixture in cases of *dysentery*, with more satisfaction, and with better success, than anything else I ever used:—℞. Mist. camph. f ℥viij.; acid nitric, fʒj.; tr. opii, fʒij. M.

As soon as I am called, whether the patient has unnatural heat or not, if an adult, I commence with ℥ss, of this mixture every three hours, and confine the patient to rice water for drink and nourishment. I sometimes add more laudanum, where the pain and tenesmus are severe. I diminish the frequency of the dose as the evacuations diminish. I frequently use no other remedy. This preparation is a modification of Hope's mixture.

No 100 Salem st., Boston, Sept. 1848. EPHRAIM BUCK

